# A big leap in prescription drug promotion in Canada

**DOI:** 10.3389/jpps.2024.12666

**Published:** 2024-01-24

**Authors:** Catherine Y. Lau

**Affiliations:** Consultant, Toronto, ON, Canada

**Keywords:** pharmaceutical advertising advisory board (PAAB), real-world evidence (RWE), Canada, patient support program (PSP), PAAB code

## Introduction

The Canadian Food and Drugs Act and Food and Drug Regulations regulate the advertising of prescription drugs in Canada. The independent not-for-profit Pharmaceutical Advertising Advisory Board (PAAB) is the preclearance agency for all promotional material distributed to healthcare professionals (HCPs) [1]. Until now, PAAB reviews have strictly complied with Health Canada-approved Product Monographs (PMs), which do not include real-world evidence (RWE) or open-label studies. For that reason, RWE and open-label studies have generally not been included in communications with HCPs [2].

In November of 2022, PAAB struck a committee to generate a framework for the acceptance of RWE in HCP advertising. The framework would focus on evidentiary standards, disclosure criteria around RWE, and other forms of evidence that do not meet gold standards but have value in clinical practice. The guidance was approved on 1 December 2023, to be launched on 1 February 2024 [3]. This announcement represents an astounding development in Canada for communicating medically important information to HCPs.

The draft RWE guidance [4] now allows pre-planned/sub-analyses of patient data matching the Canadian-approved patient population from RWE/open-label studies. This represents a big step forward in providing the timeliest information to HCPs. Inclusion of drug persistence/comparative data from patient support programs (PSPs) and market research that reflects real-life usage in Canada should provide invaluable information to HCPs.

While the PAAB guidance [4] covers important data sources, disease registries and claims databases are not mentioned. In addition, prohibiting the promotion of RWE for conditional approvals could potentially limit the communication of crucial information to HCPs. This editorial recommends additional discussion points in an attempt to improve clarity and to prepare for challenges facing the preparation and reviewing of Advertising/Promotion Systems (APS).

## Patient population and patient selection

Following the original PAAB Code, all information not consistent with a PM is considered misleading and rejected. Since it takes over 12 months to update new product claims in Canadian PMs, information in APS is generally outdated, prompting pharmaceutical companies to hire contingents of clinical liaisons to provide HCPs with updated clinical data.

The proposed RWE guidance document [4] states that “In instances where an overall study population exceeds the product’s indication, it may be possible to present data from a pre-planned patient subset that reflects the indicated patient population or relevant subset thereof.” Similarly, when preplanned, a subset of patients within an approved dose can be carved out within a bigger study consisting of patients receiving not-yet-approved dosing regimens. Information not available in PMs, such as longer-term outcomes (e.g., overall survivals for oncology, longer-term disease remission and quality of life data), can now be legitimately advertised provided that RWE used has been published in reputable peer-reviewed scientific journals (with some exceptions discussed below).

## Patient support program (PSP) and market research data

The guidance proposes that the use of RWD/RWE from PSP or market research would in certain specific cases not need to be published in peer-reviewed journals. The exception includes retention/persistence data or adherence data from the sponsor’s PSP, together with robust data capture, reporting, and analysis to validate the submission. This allowance of including unpublished PSP data in advertising has both pros and cons. Rapid communication of the most recent data to HCPs is a positive, as patients would benefit from the latest information, especially patients with cancer or chronic illness. The cons are that methodologies for the analysis of PSP data are still in rudimentary stages and a lack of peer-reviewed scrutiny will impact data validity. The burden of proof could be challenging for PAAB staff. Similarly, methodologies are needed for minimizing potential bias in retention/comparative data from market research.

## Data transparency

The guidance indicates that published RWE information must contain sufficient content for PAAB to evaluate the methodologies. As most publications have page and word limits, the best way to demonstrate complete transparency with data sources and methodologies is through the supplementals. Sponsors are advised to take advantage of supplemental sections by uploading raw data and analysis methodologies.

## Clinician assessment/acceptance

The best way to gain physician acceptance of RWE information will be to involve them throughout, from the inception of the research through the review of the analysis. Clinicians should be invited to study committees to give input, assist in monitoring, and advise data analysis in the most unbiased manner.

## Pre-planned analysis

Most real-world information would be derived from data collected retrospectively (electronic health records, PSPs, patient chart reviews, disease registries). To avoid bias, all pre-planned analyses and amendments should be pre-defined without any knowledge of databases. The use of robust analytical methods such as quantitative bias analysis (QBA) to manage confounders is recommended.

## Conflicting data

The proposed PAAB guidance also discusses publication of contradictory data from competitors. While this might be a fair and balanced way to present the information, tracking/identification of pre/post superiority data could be burdensome. PAAB might want to consider another pathway for presenting competitor data.

Canada has been considered a slow adopter of RWE for regulatory or health technology assessment [5]. On 18 December 2023, while announcing the creation of the Canadian Drug Agency (CDA) [6] to coordinate a sustainable drug system for Canadians, Minister of Health, the Honourable Mark Holland, also mentioned the creation of specific workstreams to collect pan-Canadian RWE data to support patients, inform healthcare decisions, and enable robust system analytics. This announcement coincides well with PAAB’s endorsement of the value of RWE in the upcoming guidance.
